# Assessment of atrial conduction times in patients with frequent premature ventricular complex

**DOI:** 10.1002/joa3.12806

**Published:** 2023-01-08

**Authors:** Erkan Kahraman, Nursen Keles, Kemal Emrecan Parsova, Murat Bastopcu, Mesut Karatas

**Affiliations:** ^1^ Department of Cardiology, Dr. Siyami Ersek Thoracic and Cardiovascular Surgery Training and Research Hospital University of Health Sciences Istanbul Turkey; ^2^ Department of Cardiology Zile State Hospital Tokat Turkey; ^3^ Department of Cardiovascular Surgery, Dr. Siyami Ersek Thoracic and Cardiovascular Surgery Training and Research Hospital University of Health Sciences Istanbul Turkey; ^4^ Department of Cardiology, Kartal Kosuyolu Yüksek Ihtisas Training and Research Hospital University of Health Sciences Istanbul Turkey

**Keywords:** atrial conduction time, echocardiography, premature ventricular complex

## Abstract

**Background:**

Premature ventricular complex (PVC) is a frequent finding in the general population. The atrial conduction time (ACT) is the period between the electrocardiographic P wave and the atrial mechanical contraction, and its prolongation indicates an atrial electromechanical delay (EMD). In our study, we compared atrial conduction parameters by echocardiographic methods between patients with frequent PVC and healthy control subjects.

**Methods:**

The study included 54 patients with PVC and 54 healthy volunteers. Atrial conduction parameters were measured with echocardiographic examination. The time difference between the p wave and the Am wave was measured in the septal, lateral, and tricuspid annulus regions. The interatrial EMD, left atrial intra‐atrial delay, and the right atrial intra‐atrial delay were calculated from these measurements. The groups were compared for demographic and electrocardiographic features and echocardiographic parameters.

**Results:**

Left intra‐atrial EMD, right intra‐atrial EMD, and interatrial EMD were significantly longer in the patient group (*p* = .001, *p* < .001, *p* < .001, respectively). PA lateral, PA septal, and PA tricuspid durations were significantly prolonged in the patient group (all *p* < .001). All ACT parameters were significantly prolonged in patients with PVC QRS duration of 150 ms and above (all *p* < .001). All ACT parameters were prolonged in PVCs of right ventricular origin than those of left ventricular origin (all *p* < .001). ACT parameters were prolonged in patients with a coupling interval time below 485 ms (all *p* < .001).

**Conclusions:**

Atrial conduction times are prolonged in patients with frequent PVC.

## 1 INTRODUCTION

Premature ventricular complex (PVC) is quite common and is detected in the majority of individuals with long‐term ambulatory rhythm monitoring. While the pathophysiological mechanisms of PVCs remain mostly unknown, potential mechanisms include triggered activity, increased automaticity, and reentry. Advanced age, a taller height, a history of coronary heart disease, arterial hypertension, smoking, and decreased physical activity each predict a greater PVC frequency. A higher PVC frequency is associated with an increased risk of cardiovascular death, cardiomyopathy, and ischemic stroke.^1^, ^2^, ^3^

PVC most frequently affects ventricular function in structurally normal heart. PVC can also cause retrograde ventriculoatrial conduction. These retrograde atrial activations can affect cardiac conduction systems like atrial ectopies. Increased PVC frequency correlates with increased left atrial (LA) maximum volume.^4^, ^5^, ^6^, ^7^

The atrial conduction time (ACT) represents the period between the electrocardiographic P wave and atrial mechanical contraction. The prolongation of ACT is termed the atrial electromechanical delay (EMD). Interatrial conduction times can be measured without invasive methods using Doppler and tissue Doppler imaging (TDI) in echocardiography.^8^, ^9^, ^10^

Prolonged ACT is associated with increased LA diameter and reduced LA function. It has been shown that left atrium negative remodeling may develop and LA diameter may increase in patients with frequent PVCs. Therefore prolonged ACT may be expected in patients with frequent PVCs.^12^, ^13^, ^14^, ^15^, ^16^, ^17^, ^18^

In this study, we compared atrial conduction parameters by echocardiographic methods between patients with frequent PVC and healthy control subjects.

## 2 METHODS

### 2.1 Study population

Fifty‐four patients with more than 10 000 PVC observed over 24 h of ambulatory rhythm Holter monitoring and 54 healthy volunteers with comparable demographic characteristics were included in the study. Twenty‐four hours of ambulatory rhythm Holter monitoring revealed no arrhythmia in the control group. Patients with coronary artery disease reduced left ventricle ejection fraction (LVEF 50%), other arrhythmias (such as supraventricular tachycardia and atrial flutter), moderate and severe valvular heart disease, significant congenital heart disease, or left ventricle (LV) hypertrophy were excluded from the study. Since current literature does not suggest a cutoff value for frequent premature atrial contractions (PAC), we considered frequent PAC to be present similar to frequent PVC which was defined as more than 30 PVC in an hour.^19^, ^20^ Patients with a PAC of 30 or more per hour were not included in the study. Patients with any type of chronic systemic or inflammatory illness or any form of malignancy that may impair the structure and function of the cardiovascular structure were also excluded from the study. The local ethics committee approved of the research. All individuals provided informed permission before participating in the study.

### 2.2 Electrocardiography

We assessed resting electrocardiograms. PVCs were analyzed regarding the QRS duration, amplitude, and morphology. PVCs are classified with respect to the presumed chamber of origin. In general, the left bundle branch block (LBBB) morphology of PVCs originates from the right ventricle (RV) and RVOT, however, PVCs originating from the septal left ventricular outflow tract (LVOT) or aortic cusp may also have LBBB morphology. Right bundle branch block (RBBB) morphology represents left ventricular origin. To distinguish right ventricular outflow tract (RVOT) versus LVOT origin we used precordial transition of R wave (late vs. early). A QRS transition in the precordial leads later than that in sinus rhythm suggests an RVOT exit (and vice versa). If the QRS transition in both PVC and sinus beats is at V3, the “R wave transition ratio” can provide further guidance. When comparing the PVC R‐wave amplitude in V2 with that in sinus rhythm, a ratio ≥0.6 predicts a left‐sided origin. A coupling interval is defined as the interval measured from the onset of the R wave of the previous sinus beat to the onset of the PVC.

### 2.3 Echocardiography

**FIGURE 1:**
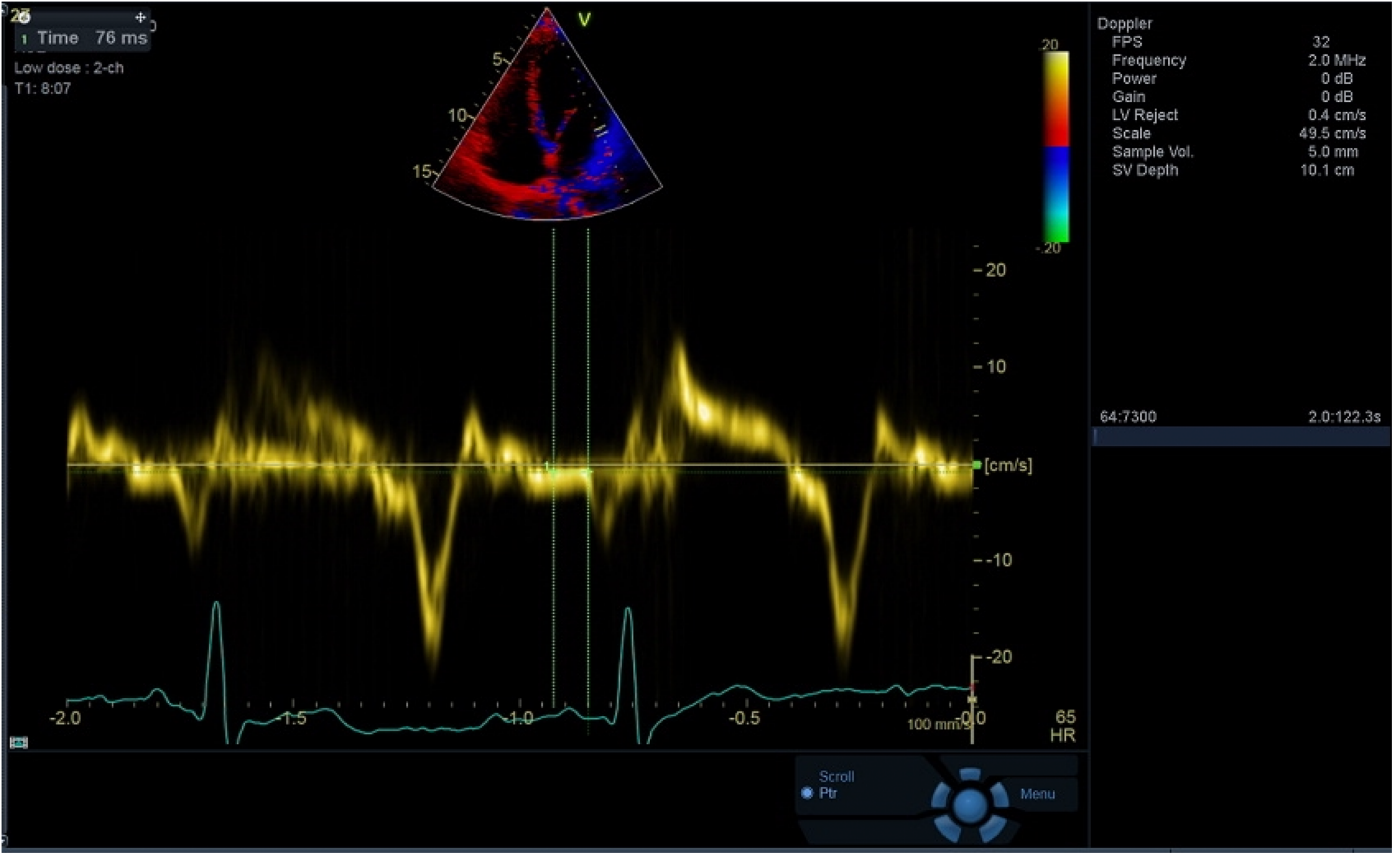
The image shows PA lateral measurement. Electrocardiogram were obtained during the examination. Am wave was obtained from the lateral mitral annulus by TDI. PA lateral was the time difference between the onset of the p wave and the onset of the lateral Am wave.

All participants were evaluated using the GE Vivid E95 system (GE Healthcare). Echocardiographic examination was performed within the first 24 h after rhythm Holter recordings were obtained. Single lead electrocardiography recording was acquired from all participants during echocardiographic assessment. The diameters of the left atrium (LA) and the LV were measured in accordance with the guidelines of the American Society of Echocardiography.^11^ The Simpson technique was utilized to determine LVEF. The TAPSE was measured using M mode from the apical four‐chamber view.^11^ The TAPSE was measured using M mode from the apical four‐chamber view. E wave, A wave, E/A ratio, isovolumic relaxation time, and E‐wave deceleration time were measured using pulsed wave Doppler from the apical four‐chamber view. The Em and Am waves of the mitral lateral, septal annulus, and tricuspid lateral annulus were obtained by TDI. PA septal was the time difference between the beginning of the p wave and the onset of the septal Am wave, PA lateral was the time difference between the onset of the p wave and the onset of the lateral Am wave, PA tricuspid was the time difference between the onset of the p wave and the tricuspid annulus Am wave (Figure 1). The interatrial EMD was the time difference between the PA lateral and the PA tricuspid (PA lateral‐PA tricuspid), the LA intra‐atrial delay was the time difference between the PA lateral and the PA septal (PA lateral‐PA septal), and the right atrial (RA) intra‐atrial delay was the time difference between the PA septal and the PA tricuspid (PA septal‐PA tricuspid).

### 2.4 Statistical analysis

Statistical analysis was performed using IBM SPSS Statistics 22 software. Nominal variables are presented as numbers and percentages and continuous variables are presented as mean and standard deviation. The chi‐squared test or Fisher's exact test was applied for nominal variables, Student's *t*‐test for continuous parametric variables, and Mann Whitney *U* test for continuous non‐parametric variables in group comparison. Univariate odds ratios (OR) were calculated for echocardiographic measurements associated with PVC and adjusted ORs were calculated controlling for other variables significant in univariate analysis. A *p* value of less than .05 was accepted as statistically significant.

## 3 RESULTS

In the study, there were 54 patients (mean age 45.9 ± 13.6 years and 40.7% male) and 54 control subjects (mean age 41.6 ± 10.4 years and 61.1% male) included. Table 1 displays the baseline demographic and clinical features of the participants. The baseline characteristics of the study groups were similar. Beta‐blockers are the most used drug group in the patient group (30 of 54 patients, 55.5%). There was no drug use in seven patients. The mean PVC frequency observed in 24‐h ambulatory rhythm Holter recordings in the patient group was 16 558 ± 7423 beats per day. The PVC of 45 patients was in the morphology of LBBB, while the PVC of nine patients was in the morphology of RBBB. The QRS duration of PVCs was calculated as 147.4 ± 11.5 ms and the coupling interval time was calculated as 496.8 ± 72.1 ms.

**TABLE 1:** Baseline demographic and clinical characteristics of the study population

	Control group (*n* = 54)	Patient group (*n* = 54)	*p* value
Age	41.6 ± 10.4	45.9 ± 13.6	.073
Gender			
Male	33 (61.1%)	22 (40.7%)	.034
Female	21 (38.9%)	32 (59.3%)	
BMI	26.22 ± 3.93	28.30 ± 4.84	.035
HT	11 (20.4%)	18 (33.3%)	.129
DM	5 (9.3%)	5 (9.3%)	1.000
HL	7 (13.0%)	8 (14.8%)	.781
Smoking	10 (18.5%)	7 (13.0%)	.428
Drug use			
No drugs		7 (12.9%)	
Beta‐blockers		30 (55.5%)	
Propafenone		6 (11.1%)	
Nondihydropyridine CCB		3 (5.5%)	
Beta‐blockers+propafenone		5 (9.2%)	
B eta‐blockers+amiodarone		3 (5.5%)	

Abbreviations: BMI, body mass index; CCB, calcium channel blockers; DM, diabetes mellitus; HL, hyperlipidemia; HT, hypertension.

Table 2 displays the echocardiographic parameters of the study group. LA anterior–posterior dimension and LA maximum volume were significantly larger in the patient group (*p* < .001 and *p* < .001 respectively). Mitral A wave velocity and E/Em ratio, and LV systolic diameter were higher in the patient group (*p* = .016, *p* = .003, and *p* = .003, respectively). The remaining echocardiographic parameters did not differ between the patient and control groups.

**TABLE 2:** Echocardiographic parameters of the study group

	Control group (*n* = 54)	Patient group (*n* = 54)	*p* value
LVEF (%)	59.87 ± 9.42	58.65 ± 4.07	.383
LVDD (mm)	4.5 ± 0.3	4.6 ± 0.3	.716
LVSD (mm)	2.7 ± 0.3	2.9 ± 0.3	**.003**
LAD‐AP (mm)	3.0 ± 0.4	3.3 ± 0.4	**<.001**
IVS (mm)	1.0 ± 0.1	1.0 ± 0.1	.174
PW (mm)	1.0 ± 0.1	1.1 ± 0.8	.730
E (cm/s)	0.70 ± 0.14	0.71 ± 0.17	.779
A (cm/s)	0.61 ± 0.13	0.67 ± 0.14	**.016**
E/A ratio	1.19 ± 0.34	1.11 ± 0.31	.228
Em (cm/s)	0.19 ± 0.20	0.14 ± 0.04	.055
Am (cm/s)	0.15 ± 0.13	0.13 ± 0.08	.850
E/Em ratio	4.57 ± 1.23	5.47 ± 1.61	**.003**
IVRT (ms)	90.5 ± 17.8	93.4 ± 12.6	.477
IVCT (ms)	90.5 ± 16.7	91.7 ± 14.7	.851
DT (ms)	154.9 ± 28.5	186.4 ± 147.3	.221
TAPSE (mm)	2.4 ± 0.3	2.4 ± 0.4	.855
LAV max (mL)	38.89 ± 9.63	46.56 ± 10.19	**<.001**

*Notes*: Bold indicates statistically significant values.

Abbreviations: A, mitral inflow late diastolic velocity; Am, mitral inflow late diastolic tissue velocity; DT, left ventricular deceleration time; E, mitral inflow early diastolic velocity; Em, mitral inflow early diastolic tissue velocity; IVCT, the isovolumic contraction time; IVRT, isovolumic relaxation time; IVS, interventricular septum thickness; LAD‐AP, left atrium anterior–posterior diameter; LAV max, left atrium maximum volume; LVDD, left ventricular diastolic diameter; LVEF, left ventricular ejection fraction; LVSD, left ventricular systolic diameter; PW, posterior wall thickness; TAPSE, tricuspid annular plane systolic excursion.

Table 3 displays the ACT intervals of the study population. In the patient group, left intra‐atrial EMD, right intra‐atrial EMD, and interatrial EMD were significantly longer (*p* = .001). PA lateral, PA septal, and PA tricuspid durations were significantly prolonged in the patient group (*p* < .001).

**TABLE 3:** Atrial conduction time intervals of the study population

	Control group (*n* = 54)	Patient group (*n* = 54)	*p* value
PA lateral (ms)	70.73 ± 17.97	90.24 ± 14.86	<.001
PA septal (ms)	70.97 ± 17.87	90.43 ± 13.65	<.001
PA tricuspid (ms)	71.13 ± 17.80	89.96 ± 15.12	<.001
Intra‐LA EMD (ms)	23.0 ± 27.0	43.9 ± 56.1	.001
Intra‐RA EMD (ms)	21.1 ± 23.3	47.9 ± 40.6	<.001
Interatrial EMD (ms)	24.4 ± 26.9	48.9 ± 40.4	<.001

Abbreviations: EMD, electromechanical delay; LA, left atrium; RA, right atrium.

**FIGURE 2:**
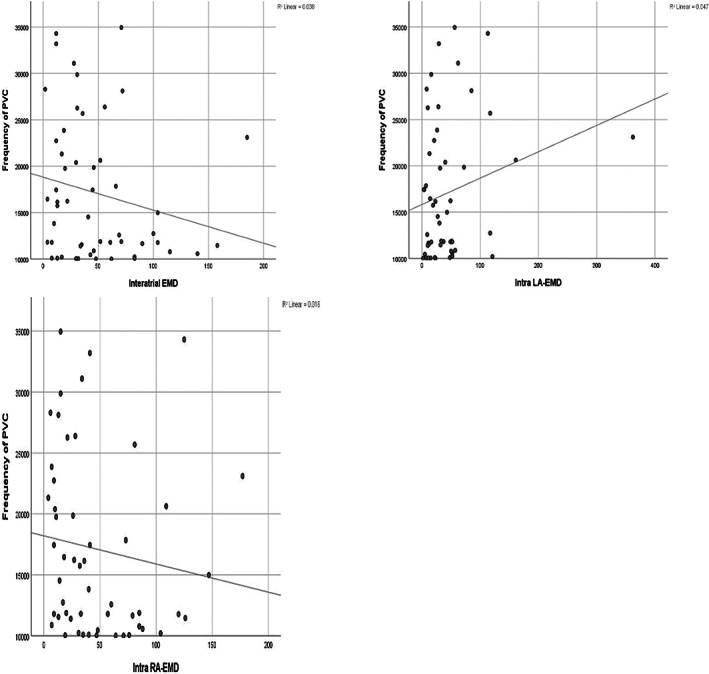
Scatter plots with a correlation coefficient that show no relationships between each ACT parameter and frequency of PVCs.

**FIGURE 3:**
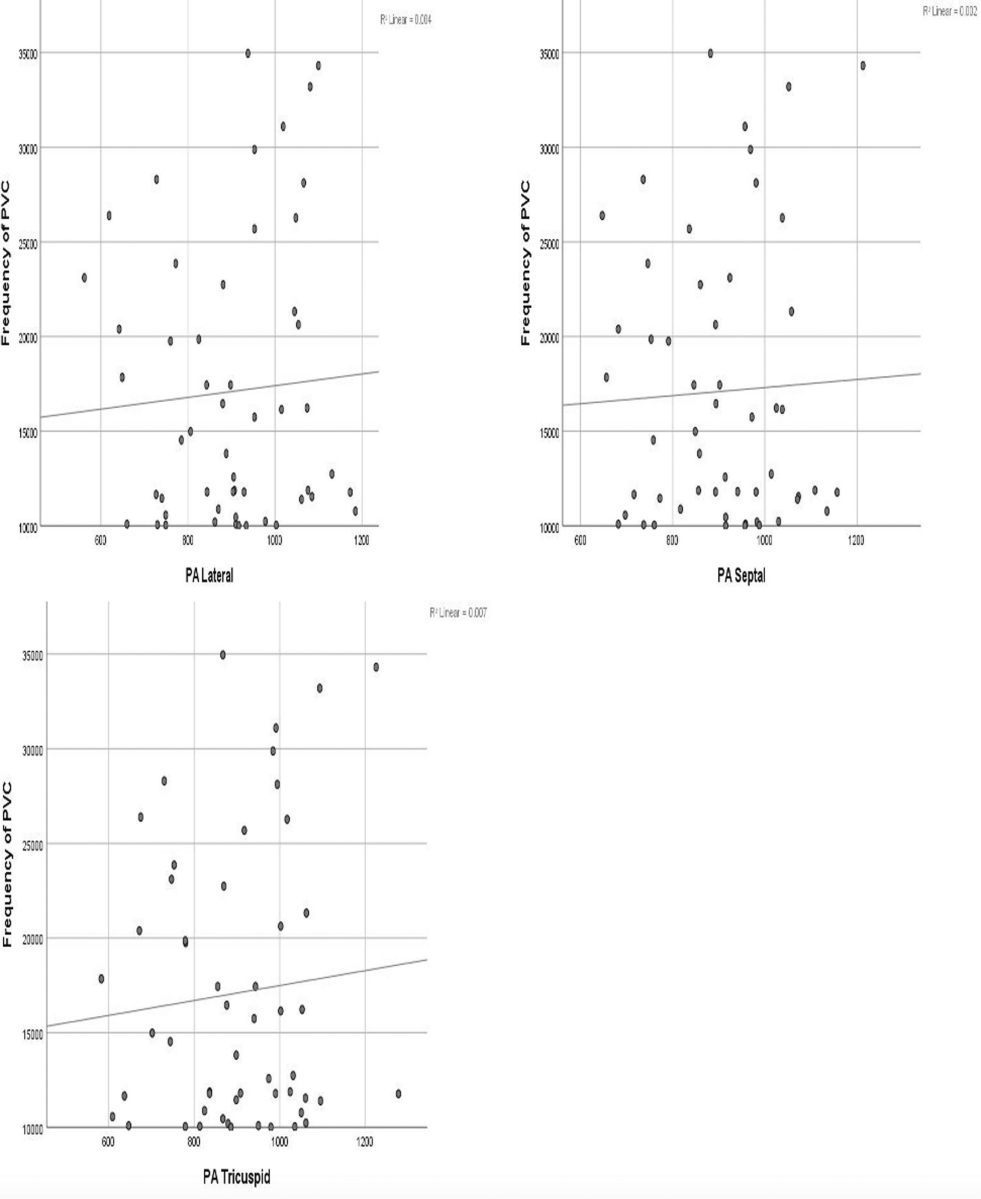
Scatter plots with a correlation coefficient that show no relationships between each ACT parameter and frequency of PVCs.

Univariate OR was calculated for each conduction time interval. The effects of conduction time intervals on PVC were adjusted for gender, BMI, LVSD, LAD‐AP, A, and E/Em which were significant in univariate analysis. The studied conduction time intervals remained significant for the presence of PVC after adjustment and the ORs are presented in Table 4. Figures 2 and 3 demonstrate scatter plots with a correlation coefficient that shows the no relationship between each ACT parameter and frequency of PVCs.

**TABLE 4:** Univariate and adjusted odds ratios of variables associated with premature ventricular complex

	Univariate OR	*p*	Multivariate OR	*p*
PA lateral (ms)	1.007 (1.004–1.010)	<.001	1.007 (1.004–1.010)	<.001
PA septal (ms)	1.008 (1.004–1.011)	<.001	1.007 (1.004–1.011)	<.001
PA tricuspid (ms)	1.007 (1.004–1.010)	<.001	1.007 (1.003–1.010)	<.001
Intra‐LA EMD (ms)	1.017 (1.003–1.031)	.001	1.021 (1.002–1.040)	.027
Intra‐RA EMD (ms)	1.029 (1.013–1.045)	<.001	1.040 (1.018–1.063)	<.001
Interatrial EMD (ms)	1.024 (1.010–1.039)	<.001	1.027 (1.009–1.046)	.003

Abbreviations: EMD electromechanical delay; LA, left atrium, OR, odds ratio; RA, right atrium.

All ACT parameters were significantly prolonged in patients with PVC QRS duration of 150 ms and above, compared to patients with PVC QRS duration below 150 ms (all *p* < .001). It was observed that all ACT parameters were prolonged in PVCs of right ventricular origin than those of left ventricular origin (all *p* < .001). ACT parameters were prolonged in patients with a coupling interval time below 485 ms (all *p* < .001) (Table 5).

**TABLE 5:** Subgroup analysis according to PVC characteristics of the patient group

	Probable left ventricle origin (*n* = 12)	Probable right ventricle origin (*n* = 42)	*p* value
PA lateral (ms)	71.68 ± 7.24	95.54 ± 11.91	<.001
PA septal (ms)	73.68 ± 5.01	95.21 ± 11.34	<.001
PA tricuspid (ms)	71.97 ± 7.09	95.10 ± 12.66	<.001
Intra‐LA EMD (ms)	8.5 ± 3.4	54.1 ± 59.9	<.001
Intra‐RA EMD (ms)	11.2 ± 4.0	58.4 ± 40.2	<.001
Interatrial EMD (ms)	10.9 ± 4.6	59.7 ± 39.5	<.001

	QRS duration < 150 ms (*n* = 23)	QRS duration ≥ 150 ms (*n* = 31)	*p* value
PA lateral (ms)	76.40 ± 9.23	100.51 ± 8.46	<.001
PA septal (ms)	77.52 ± 7.52	100.00 ± 8.09	<.001
PA tricuspid (ms)	76.00 ± 9.01	100.32 ± 9.18	<.001
Intra‐LA EMD (ms)	11.6 ± 6.0	67.9 ± 64.3	<.001
Intra‐RA EMD (ms)	14.9 ± 7.0	72.5 ± 37.6	<.001
Interatrial EMD (ms)	16.0 ± 9.0	73.2 ± 37.2	<.001

	Coupling interval < 485 ms (*n* = 27)	QRS duration ≥ 485 ms (*n* = 27)	*p* value
PA lateral (ms)	102.01 ± 8.02	78.46 ± 9.88	<.001
PA septal (ms)	101.47 ± 7.61	79.38 ± 8.29	<.001
PA tricuspid (ms)	102.05 ± 8.54	77.87 ± 9.47	<.001
Intra‐LA EMD (ms)	74.1 ± 66.8	13.7 ± 7.6	<.001
Intra‐RA EMD (ms)	78.4 ± 36.7	17.5 ± 9.1	<.001
Interatrial EMD (ms)	79.0 ± 36.4	18.7 ± 10.6	<.001

Abbreviations: EMD, electromechanical delay; LA, left atrium; RA, right atrium.

## 4 DISCUSSION

Patients with frequent PVC had prolonged ACTs in our study as assessed by PA lateral, PA septal, PA tricuspid, intra‐LA EMD, intra‐RA EMD, and interatrial EMD. Atrial EMD was studied before in patients with frequent PVCs in the study by Gurbuz et al. where 21 patients with frequent RVOT PVC were evaluated. Their study showed prolonged LA conduction times in patients with frequent RVOT PVC and preserved LVEF.^9^ In our study, in which more patients with frequent PVC were evaluated, we observed prolongation of the LA conduction time, as well as the prolongation of the right atrial and interatrial conduction times.

Prolonged ACT is found to be associated with increased LA diameter and reduced LA systolic function, therefore atrial EMD reflects structural and electrophysiological remodeling of the LA. There are common risk factors between prolonged ACT and PVC, such as advanced age, hypertension, and heart failure. Age‐related fibrosis can cause prolonged ACT, and LA dilation. Other well‐established risk factors are hypertension and heart failure. Hypertension or heart failure can lead to LA remodeling by volume overload and increased atrial pressure, and diastolic dysfunction can also lead to atrial dilatation and fibrosis.^12^, ^13^, ^14^, ^15^ Additionally, prolonged ACT may be related to an effect of inflammatory cytokines in AF patients. Systemic inflammation can cause subclinical cardiac damage. Some inflammatory disorders, such as psöriazis and ulcerative colitis, are shown to be associated with atrial EMD prolongation.^16^, ^17^, ^18^

Previous studies demonstrated larger LA sizes in patients with frequent PVC. The burden of PVC showed an independent association with LA enlargement in patients with frequent PVCs and preserved LVEF.^7^ PVC causes electrical and mechanical dyssynchrony because of which the atria may contract against the closed mitral valves. In addition, as the PVC QRS duration increases, atrioventricular dyssynchrony increases. A longer PVC duration may relationship to greater dyssynchrony in LV contraction and is associated with impaired LV function.^21^ This dyssynchrony increases LA pressure and an increase in LA wall stress. With this mechanism, frequent PVC can lead to unfavorable remodeling of the atria.^7^, ^22^, ^23^ Prolongation of ACTs may occur as a result of such remodeling and an increase in E/Em ratios and A velocities are observed in the echocardiography of patients with frequent PVCs. Although the E/Em ratio (5.47 ± 1.61) and A wave (0.67 ± 0.14) were higher in the patient group, the values did not meet the diastolic dysfunction criteria.

PVCs originating from the RV may result in a more serious reduce in LVEF than those coming from the LV. A short coupling interval may also result in considerably reduced LV stroke volumes because of less LV filling time, but the short coupling interval by itself does not relationship between LV dyssynchrony.^24^ An impaired LVEF will likewise have significant effects on LA functioning. In our study, ACT parameters were considerably prolonged in patients with PVC originating from the RV and in patients with short coupling interval.

The progression of PVC to AF is not clearly illustrated, but a higher frequency of new‐onset AF has been shown in patients with frequent PVC. During PVC, retrograde ventriculoatrial activation may be triggered which acts as an atrial ectopy. Frequent atrial ectopia can lead to impaired ACT. It is possible that PVC increases the risk of AF development via retrograde ventriculoatrial conduction, impaired LA function and remodeling and impaired ACT.^4^, ^25^ Studies with longer follow‐up times are needed to investigate the association of prolonged atrial conduction times and new‐onset AF in patients with PVC.

## 5 STUDY LIMITATIONS

The study included a relatively small group of patients for the patient and control groups. Electrophysiological study (EPS) was not performed for PVC evaluation and determination of origin. Many other common risk factors may lead to prolonged ACT in patients with PVCs such as older age and hypertension. Further study is necessary to understand these interactions clearly. Patients were not followed up in the long term for the onset of AF. Studies with a larger number of participants and longer follow up are needed to investigate the development of AF in patients with frequent PVC.

## 6 CONCLUSIONS

Atrial conduction times are prolonged in patients with frequent PVC.

## CONFLICT OF INTEREST

The work will be presented at the “European Society of Cardiology (ESC) Congress 2022, Barcelona” as an oral moderated poster presentation and will be published as an abstract in the supplement of the European Heart Journal.

## ETHICAL APPROVAL

This study was approved by the ethics committee of the University of Health Sciences, Haydarpasa Numune Training and Research Hospital, Turkey (Approval Code: HNEAH‐KAEK 2020/146), and the research protocol followed the guidelines of the Declaration of Helsinki.

## PATIENT CONSENT

All participants provided informed consent before participating in the study.

## Data Availability

The authors confirm that the data supporting the findings of this study are available within the article.
